# Comparative Evaluation of Low-Level Laser Therapy and Topical Triamcinolone Acetonide 0.1% in Recurrent Aphthous Stomatitis Subjects

**DOI:** 10.7759/cureus.25564

**Published:** 2022-06-01

**Authors:** Ashwini Dhopte, Hiroj Bagde

**Affiliations:** 1 Oral Medicine and Radiology, Rama Dental College and Research Centre, Kanpur, IND; 2 Periodontology, Rama Dental College and Research Centre, Kanpur, IND

**Keywords:** ­wound healing, pain, triamcinolone acetonide, aphthous stomatitis, low level laser therapy

## Abstract

Background

Recurrent aphthous stomatitis (RAS), sometimes known as canker sores, is an infection of the oral mucous membranes caused by an inflammatory process. Triamcinolone and low-level laser therapy for recurrent aphthous ulcers were studied in this research, which evaluated their clinical efficacy.

Methodology

Among 60 subjects, 54 subjects fulfilled the inclusion criteria with an age range between 16 and 46 years. Subjects were randomly divided into two groups, the control group who received four times daily topical application of triamcinolone acetonide 0.1% and the test group who underwent a single application of low-level laser therapy on three alternate days. Evaluations were done on day 1, day 3, day 5, and day 10. Data obtained on oral-health-related quality of life (OHR-QoL) using the OHR-QoL questionnaire were used as baseline data. Clinical parameters like pain score were assessed using a visual analog scale, ulcer size using UNC-15 probe, and erythema and healing score were evaluated using erythema and healing scale.

Result

According to the OHR-QoL data, there was no statistically significant difference in the quality of life of test and control subjects. We found that pain and ulcer size reduced considerably (p=0.007) from the first day to the third day (p=0.000), and then dropped significantly again (p=0.000) on days 3-10 (p=0.000). On days 3 and 5, the test group showed significant reductions in pain and erythema (p=0.13), as well as an improvement in ulcer healing when compared to the control group. On days 3 and 5, the size of the ulcers decreased similarly in the control and test groups. On the 10th day, patients in both groups had less discomfort, erythema, and ulcer size, as well as improved healing of the ulcer.

Conclusion

Pain and erythema reduction and ulcer healing improvement were significantly higher in subjects in the test group as compared to those in the control group by day 3. The reduction in ulcer size was comparable to the control group on days 3 and 5, while the complete reduction in ulcer size was seen in both groups by day 10.

## Introduction

Recurrent aphthous stomatitis (RAS), sometimes known as canker sores, is an infection of the oral mucous membranes caused by an inflammatory process [1]. One of the most common manifestations in infancy and adolescence is the development of painful, tiny, round or oval ulcers with a defined boundary, erythematous haloes, and yellow or grayish floors [2]. It is thought that T-lymphocytes have a crucial role in the development of this kind of immunological dysfunction. Many etiological variables, such as genetics, immunology, hypersensitivity to foods and medications, hormonal changes, trauma, hematological deficiencies (serum iron, folate, and vitamin B12), environmental and psychological stress, and viral infections, are indicated [3].

RAS is divided into three distinct categories: minor, major, and herpetiform. The majority of minor ulcers are smaller than 1 centimeter in diameter, last for 7-14 days, and heal completely without leaving any scars behind. More than a centimeter in diameter, a major ulcer typically heals in 20-30 days, and scarring is common. Lesions are many, grouped up to 3 millimeters in diameter, and they normally heal in 15 days [4].

Management focuses on relieving the patient's symptoms of discomfort. Therapy agents such as topical steroids, oral tetracycline (tetracycline mouth rinse), silver nitrate cautery (low-level laser therapy [LLLT]), and cryotherapy have all been used to relieve pain in patients [4].

The etiology and causation of RAS are still a mystery. The tendency to form RAS is influenced by a wide range of variables, including genetic predisposition, psychological stress and anxiety, immunological abnormalities, local trauma, microbial agents, and hematinic insufficiency [2]. As many as 40% of patients had RAS in their family history, which clearly suggests a genetic propensity for aphthous ulcers. Anesthetic injections, dental procedures, and toothbrush injuries have all been linked to the emergence of aphthous in the past. Vitamin B12 and folic acid deficiencies may further increase the risk of developing RAS. For example, Crohn's illness and ulcerative colitis are both connected with aphthae, according to research.

Unfortunately, there is no therapy for RAS. Symptomatic alleviation is the primary goal of treatment, which aims to reduce discomfort, decrease the size and number of ulcers, and shorten the healing period. Patients may choose from a variety of medicines to treat their ailments, including antiseptic and anti-inflammatory treatments, antibiotics, and corticosteroids. Corticosteroids such as hydrocortisone hemisuccinate, triamcinolone acetate, and beclomethasone dipropionate are topical. It has anti-inflammatory and particular inhibitory effects on T-lymphocytoepithelial cells [5]. Corticosteroids, such as triamcinolone acetate, may be applied to the skin or applied to the mouth to treat RAS.

Lasers have made significant inroads into the medical sector in the last several years. Lasers, on the other hand, have been utilized in dentistry for quite some time now. LLLT is a light source therapy that provides monochromatic and coherent light of a single light wavelength. 

It works by photobiological or biochemical stimulation of cells. It changes the activities of cells and tissues. It increases ATP (adenosine triphosphate) production and reduces cellular oxygen demand by stimulating mitochondria. Serotonin and endorphin levels rise, prostaglandin E2 and interleukin-1 beta levels fall, and pain is reduced as a result [6]. Plasminogen activator, which is responsible for collagen breakdown and consequently promotes proliferation, is inhibited to lessen inflammatory response. Arterioles dilate as a result of the drug's microcirculation-enhancing properties. As a result of boosting the number of fibroblasts that proliferate and mature into myofibroblasts as well as the amount of fibroblastic growth factor secreted, it reduces the healing time. LLLT decreases edema, alleviates pain, and promotes tissue regeneration [7].

When it comes to treating people with mild RAS, this study aimed to assess the effects of LLLT and triamcinolone acetonide (TA) (0.1%).

## Materials and methods

A total of 60 patients from the outpatient department (OPD) with a recurrent aphthous ulcer and students and employees of NHDCRI were recruited/enrolled in the study. As part of the study, which lasted from December 2016 to April 2018, the New Horizon Dental College Institutional Review Board accepted the research (IRB certificate number: NHDC/PG/23/11/16). A thorough explanation of the operation and its associated risks were provided to each patient, and their informed consent was obtained. Patients were chosen at random from a list of those who had previously been tested, and of the 60 who started therapy, 54 finished it. They were split into two groups, as shown below:

Group 1 - Control subjects including 26 subjects receiving TA 0.1% topical application.

Group 2 - Test subjects with 28 subjects undergoing LLLT chair-side application.

Inclusion criteria

Patients with an authentic history of minor RAS (size lesser than 10 mm) presenting to the OPD within a day of appearance of the ulcer were included. Patients falling into the age group of 15-50 years were included, with no discrimination between the gender of the patients. Patients who were presently undergoing or had previous history of treatment for RAS were also included.

Exclusion criteria

The exclusion criteria were pregnancy and lactation, patients with artificial pacemakers as prolonged contact with electromagnetic radiation and laser can affect the working of the pacemaker, patients with malignant diseases, any history of systemic diseases, such as HIV and diabetes mellitus, or any condition that could predispose to oral ulceration, and patients having ulcers that are caused due to topical or systemic medications and ulcers currently under treatment with any topical medication or corticosteroids.

Procedure

Subjects enrolled into the study were screened to exclude ulcers due to any other causes or secondary to systemic diseases. On the initial visit, all patients were asked to fill out a questionnaire that included demographic information, such as age and gender, as well as specific questions concerning the disease, such as the presenting complaint, the first episode of ulcer, the frequency, and the number of ulcers. OHR-QoL14 (overall health-related quality of life appraised by OHIP-14) English/Hindi questions asked to the respondents included how often they had encountered RAS to influence their quality of life in the recent month. Likert scale responses were classified as follows: 4 (often), 3 (frequently), 2 (sometimes), 1 (rarely), and 0 (never). Summarizing the responses to the 14 questions, a scale with higher scores indicating worse OHR-QoL was used to get the final score [8,9].

From day 1 through day 5, the participants were instructed to abstain from consuming any topical or systemic drugs or items, even if they had previously used such treatments.

By tossing a coin, the subjects were randomly allocated into the control and test groups upon presentation with symptoms and newly formed oral ulcers of RAS.

Group 1 subjects received the topical TA 0.1%. Primary application was done by the clinician at the chairside and the patient was educated about the application, dosage, and frequency. A little amount of gel was applied to the ulcer four times a day and left on the ulcer for half an hour before eating or drinking anything. We used the same measuring applicator on every single patient. The patients were asked to apply the gel daily from day 1 to 5 . There was a 10-day follow-up period for the subjects. A compliance chart was maintained by the patients.

In group 2, there was a diode laser used to deliver LLLT to the participants, with all appropriate laser safety precautions in place. An 810 nm wavelength, 6.3 J/cm diffraction, defocused mode laser was used, beginning at 10 mm from the lesion and gradually approaching the lesion within a range of 2 mm for roughly 30 seconds for initial passage. To gauge how much pain a patient was feeling, researchers let him or her take sips of water. Two extra 30-second passes were used since the patient had not reported any meaningful improvement in discomfort during the first two rounds. Between each pass, a 20-second cooling period was supplied, and the total laser exposure time was less than 2 minutes. LLLT was given on baseline, day 1, day 3, and day 5. There was a 10-day follow-up period for the subjects.

The ulcers were evaluated by the following criteria: pain [1] - In order to gauge the intensity of pain, the participants utilized a 10-point visual analog scale, with 0 representing no pain, 1-5 representing moderate discomfort, and 6-10 representing severe discomfort; ulcer size [1] -The measurement of lesion size was done in each visit using calibrated UNC-15 probe; erythema scale [10] - The presence, decrease, and absence of erythema were evaluated using the criteria defined in Table 1; and healing scale [8] - Improvement in healing of ulcer was assessed using the criteria defined in Table 2.

**Table 1:** Erythema score index

Score	Criteria
0	No erythema
1	Light red/pink
2	Red but not dark in color
3	Very red/dark in color

**Table 2:** Healing index EI: erythema index

Score	Criteria
0	No improvement: EI <30
1	Moderate improvement: 70%>EI≥30%
2	Marked improvement: 100%>EI≥70%
3	Heal: EI=100%

Evaluation was done on days 1, 3, 5, and 10.

Statistical analysis

An Excel spreadsheet was used to generate and tabulate the data, and Statistical Package for Social Science (SPSS) version 21 (IBM Corp, Armonk, NY) was used for statistical analysis. There were paired t-tests and one-way analysis of variance (ANOVA) tests used to compare the findings across groups and individual visits, while Wilcoxon tests, Mann-Whitney tests, and chi-square tests were used to examine the healing of erythema and ulcers. In this investigation, we considered significance to be a p-value of less than 0.05.

## Results

In Bilaspur, Chhattisgarh, from December 2016 to April 2018, researchers from the NHDCRI's department of oral medicine and radiology examined patients. Twenty-two men and 48 women took part in the research. There were two more groups of participants: the control group got TA 0.1% topical treatment, while the test group received LLLT, with a total of 60 individuals in total.

“There were eight men and 18 women in the control group, and 11 men and 17 women in the test group. The mean age was 28.13 years in the control group and 25.67 years in the test group for both groups of research participants. The age range for both groups was between 17 and 46 years of age. The mean age of the control and test respondents did not vary significantly.”

Quality of life

With regard to the quality of life, the control subjects had a mean difference of 18.7931 ± 6.6352 as compared to the test subjects having a mean difference of 20.7742± 7.4953. It was found to be statistically not significant (p=0.284) (Table 3).

**Table 3:** Quality of life among control and test groups NS: not significant

Group	N	Mean difference	Standard deviation	F-value	p-Value	Significance
Control	29	18.7931	6.63529	1.169	0.284	NS
Test	31	20.7742	7.49537			

Using individual questions from the overall sample, it was found that 81.7% had difficulty pronouncing any words, followed by 90% who thought their sense of taste had deteriorated, 98.3% who experienced terrible hurting in the mouth, and 95% who found it difficult to chew any food. A total of 68.3% of the participants reported feeling self-conscious and 63.3% reported feeling tense because of a problem with their teeth or mouth; diets were unsatisfactory for 83.3% and 90% had to interrupt meals because of teeth or mouth problems; 78.3% reported difficulty relaxing, 71.7% found themselves embarrassed, and 70% were irritable with other people (Table 4).

**Table 4:** Questionnaire about quality of life

S.no	Quality-of-life questions	Frequency	Percentage
1	Have you had trouble pronouncing any words because of problems with your teeth or mouth?	49	81.7
2	Have you felt that your sense of taste has worsened because of problems with your teeth or mouth?	54	90
3	Have you had painful aching in your mouth?	59	98.3
4	Have you found it uncomfortable to eat any food because of problem with your teeth or mouth?	57	95
5	Have you been self-conscious because of your teeth or mouth?	41	68.3
6	Have you felt tense because of problems with your teeth or mouth?	38	63.3
7	Has your diet been unsatisfactory because of problems with your teeth or mouth?	50	83.3
8	Have you had to interrupt meals because of problems with your teeth or mouth?	54	90
9	Have you found it difficult to relax because of problems with your teeth or mouth?	47	78.3
10	Have you been a bit embarrassed because of problems with your teeth or mouth?	43	71.7
11	Have you been a bit irritable with other people because of problems with your teeth or mouth?	42	70
12	Have you had difficulty doing your usual job because of problems with your teeth or mouth?	36	60
13	Have you felt that life in general was less satisfying because of problems with your teeth or mouth?	38	63.3
14	Have you been totally unable to function because of problems with your teeth or mouth?	29	48.3

Pain

There was no statistically significant difference in pain levels across groups (p=0.104). After three days, the test group showed a statistically significant reduction in pain; however, this difference was minor (p=0.798). No significant difference was seen between the test and control groups on the fifth day (p=0.853) in the reduction of pain ratings. On day 10, the test group showed slightly more pain score reduction (0.0714±0.26227) than the control group (0.0769±0.27175) but was statistically nonsignificant (p=0.940) (Table 5).

**Table 5:** Change in pain score from the first visit to the fourth visit within group VAS: visual analog scale

Parameter	Control			Test			F-value	p-Value
	Mean (VAS)		Standard deviation	Mean (VAS)	Standard deviation			
Pain score 1 day	4.5000	1.79444		5.3214		1.84699	2.74	0.104
Pain score 3 days	2.9231	1.64738		3.0714		2.47848	0.066	0.798
Pain score 5 days	0.8077	1.02056		0.7500		1.23603	0.035	0.853
Pain score 10 days	0.0769	0.27175		0.0714		0.26227	0.006	0.940

Ulcer size

Ulcer size did not vary between the test and control groups statistically (p=0.316) in the whole sample. In terms of reduction in ulcer size, there was no statistically significant difference between the two groups on day 3 (p=0.651). Neither the test nor the control groups (p=0.967) revealed a significant difference in ulcer size reduction on day 5. On day 10, the test group and control group showed a statistically significant difference in ulcer size reduction (Table 6).

**Table 6:** Change in ulcer size from the first visit to the fourth visit within group

Parameter	Control		Test		F-value	p-Value
	Mean (ulcer size)	Standard deviation	Mean (ulcer size)	Standard deviation		
Ulcer size 1 day	2.8077	1.41476	3.1786	1.27812	1.024	0.316
Ulcer size 3 days	2.1154	1.27521	2.2857	1.46204	0.207	0.651
Ulcer size 5 days	0.8462	0.92487	0.8571	1.00791	0.002	0.967
Ulcer size 10 days	0.0000	0.00000	0.0000	0.00000		

Erythema score

On day 1, all subjects had some form of erythema. Moderate erythema was seen in 42.3% (N=11) of control subjects as compared to 39.3% (N=11) of test subjects. Marked erythema was seen in 50% (N=13) of control subjects as compared to 53.6% (N=15) of test subjects. Dark erythema was seen in 7.7% (N=2) of control subjects as compared to 7.1% (N=2) of test subjects. No statistical difference existed between both the groups (p=0.88). On day 3, no erythema was seen in 3.8% (N=1) of control subjects as compared to 28.6% (N=8) of test subjects. Moderate erythema was seen in 69.2% (N=18) of control subjects as compared to 46.4% (N=13) of test subjects. Marked erythema was seen in 26.9% (N=7) of control subjects as compared to 25% (N=7) of test subjects. A statistically significant difference (p=0.13) existed between both the groups since the test group showed a higher percentage of subjects with no erythema (28.6%). On day 5, all subjects showed a reduction in erythema, 50% (N=13) of subjects showed no erythema in the control group as compared to 60.7% (N=17) of test subjects. Moderate erythema was seen in 50% (N=13) of control subjects as compared to 39.3% (N=11) of test subjects. A statistically significant difference (p=0.43) existed between both the groups since the test group showed a higher percentage of subjects with erythema reduction as compared to the control group. On day 10, a higher percentage of control subjects showed no erythema, 96.2% (N=25), as compared to 92.9% (N=26) of test subjects. Moderate erythema was seen in 3.8% (N=1) of control subjects as compared to 7.1% (N=2) of test subjects. However, no statistical difference existed between both the groups (p=0.6) (Tables 7, 8).

**Table 7:** Comparison of erythema score from the first visit to the fourth visit within the group NS: not significant; S: significant

Parameter	No erythema		Light erythema		Erythema but not dark erythema		Dark erythema		p-Value	Significance
	Control	Test	Control	Test	Control	Test	Control	Test		
Day 1			42.3%	39.3%	50.0%	53.6%	7.7%	7.1%	0.966	NS
Day 3	3.8%	28.6%	69.2%	46.4%	26.9%	25.0%			0.045	S
Day 5	50.0%	60.7%	50.0%	39.3%					0.429	NS
Day 10	96.2%	92.9%	3.8%	7.1%					0.597	NS

**Table 8:** Change in erythema score from the first visit to the fourth visit within the group

Parameter	Control		Test		T-value	p-Value
	Mean (erythema)	Standard deviation	Mean (erythema)	Standard deviation		
Erythema 1 day	1.65	0.63	1.68	0.61	-0.146	0.88
Erythema 3 days	1.23	0.5	0.96	0.74	1.519	0.13
Erythema 5 days	0.5	0.5	0.39	0.5	0.781	0.43
Erythema 10 days	0.04	0.19	0.07	0.26	-0.52	0.6

Ulcer healing score

On day 3, all subjects showed some form of improvement. No improvement was seen in 14.3% (N=4) test subjects, and moderate improvement was seen in 73.1% (N=19) of control subjects as compared to 25% (N=7) of test subjects. Marked improvement was seen in 26.9% (N=7) of control subjects as compared to 53.6% (N=15) of test subjects and only 7.1% (N=2) of test subjects showed complete healing. A statistically significant difference (p=0.15) existed between both the groups as a higher percentage of test subjects showed healing. On day 5, moderate improvement was seen in 15.4% (N=4) of control subjects as compared to test subjects. Marked improvement was seen in 46.2% (N=12) of control subjects as compared to 57.1% (N=16) of test subjects. Complete healing was seen in 38.5% (N=10) of control subjects as compared to 42.9% (N=12) of test subjects. A statistical difference existed (p=0.24) between both the groups as the test group showed a higher percentage of subjects with ulcer healing improvement as compared to the control group. On day 10, a marked improvement was seen in 3.8% (N=1) of control subjects as compared to 7.1% (N=2) of test subjects. Complete healing was seen in a higher percentage of control subjects, 96.2% (N=25), as compared to 92.9% (N=26) of test subjects. A p-value =0.6 showed that there was no statistical difference between the two groups (Tables 9, 10).

**Table 9:** Comparison of healing score from the first visit to the fourth visit within the group NS: not significant; S: significant

Parameter	No improvement		Moderate improvement		Marked improvement		Healing		p-Value	Significance
	Control	Test	Control	Test	Control	Test	Control	Test		
Day 1	100.0%	100.0%								
Day 3	0.0%	14.3%	73.1%	25.0%	26.9%	53.6%	0.0%	7.1%	0.002	S
Day 5			15.4%	0.0%	46.2%	57.1%	38.5%	42.9%	0.096	NS
Day 10					3.8%	7.1%	96.2%	92.9%	0.597	NS

**Table 10:** Change in healing score from the second visit to the fourth visit within the group

Parameter	Control		Test		T-value	p-Value
	Mean (healing)	Standard deviation	Mean (healing)	Standard deviation		
Healing 3 days	1.27	0.45	1.54	0.83	-1.43	0.15
Healing 5 days	2.23	0.7	2.43	0.5	-1.18	0.24
Healing 10 days	2.96	0.19	2.93	0.26	0.52	0.6

The most frequent kind of recurrent aphthous ulcer affects 5-66% of people. It is more frequent in those between the ages of 10 and 40, and women and people with better socioeconomic status are more likely to be affected. On the non-keratinized mucosa of the mouth, ulceration may cause problems eating, speaking, and swallowing. Anywhere from 2 to 48 hours before an ulcer forms, the lesions begin with prodromal burning. A little region of erythema appears in the early stages. White papules appear quickly and grow in size over the following 48-72 hours before becoming an ulcer [5].

## Discussion

In the present study, aphthous ulcers were more frequently seen in females when compared with males (females=69.2%, male=30.8%) in the control group and (females=60.7%, males=39.3%) in the test group. Similar results were found by Lalabonova and Daskalov [11] (male=17.2%, female= 82.8%), while Aggarwal H et al. [12] showed male predominance (male=18, female=12). The mean age of subjects in the present study was 28.13±7.62 years, 25.67±5.27 years in the control and test groups, respectively, which was in concordance with the finding by Salman H et al. [5] (11-45 years). Lalabonova and Daskalov [11] showed a higher mean age of 43.01±1.25 years in their study similar to the present study.

The majority of subjects who participated in the present study were students. Students show a higher level of stress during exam times, and many adults also suffer when they are stressed for many days [13]. It has also been suggested that sleep and dietary insufficiency due to lifestyle choices often leads to a deficiency of essential vitamins and minerals, causing mouth ulcers [14]. 

Stress is considered to be the most common factor often predisposing to an aphthous ulcer. Psychometric evaluation through various tools are available: stress, anxiety, and depression are evaluated using a general health questionnaire (GHQ) and hospital anxiety depression (HAD) and quality of life by OHR-QoL and oral health impact profile (OHIP). In the present study, the OHR-QoL questionnaire was used with due permission for using tools. Only 81.7% had difficulty pronouncing any words, 90% thought their sense of taste had deteriorated, 98.3% experienced discomfort in their mouth, and 95% found it difficult to consume any meal when asked about their quality of life. Diets were unsatisfactory in 83.3% of patients, meals were interrupted because of teeth or mouth issues in 90%, 71.7% found themselves a little embarrassed, and 70% were a little irritable with others. Sixty percent reported difficulty in doing their usual job, and 63.3% felt that life, in general, was less satisfying. Rajan B et al. [14] showed that 51.98% suffered pain and functional impairment, whereas 57% had social and emotional problems. Sixty-eight percent of Gallo Cde B et al.'s [15] participants reported experiencing emotional stress.

Therapeutic options for aphthous ulcers range from topical steroids, antibiotics, local anesthetic gels, and silver nitrate cautery to LLLT and cryotherapy, as well as additional treatment methods such as silver nitrate cautery [16]. Reduced pain, smaller ulcers, fewer erythema flare-ups, faster healing, and lower recurrence rates are all primary therapy goals.

Low-level laser therapy

Only 42.3% of the individuals tested in the current research showed a significant decrease in pain score (p=0.000) after receiving LLLT in defocused mode (810 nm wavelength, 80 seconds per treatment, 6.3 J/cm) on day 3. On day 5, 85.9% (p=0.000) of the individuals exhibited pain reduction, and on day 10, 98.6% of the subjects showed pain reduction (p=0.000). By day 5 of treatment, the patient's overall pain score had decreased significantly (p=0.000), leading to a total reduction in pain. For example, Khademi and Am Shirani [17], de Souza et al. [18], and Ghali and Abdulhamed [19] found similar findings using a diode laser (670 nm, 50 Mw, 1.9 cm for 1 minute) and reported that 75% of patients exhibited a decrease of pain following treatment, and entire regression occurred in an average of four days in 40%.

LLLT's pain reduction mechanism is explained by increased ATP production in neurons' mitochondria. To lessen the threshold for an action potential to be initiated, the amount of ATP being synthesized is decreased. A reduction in pain stimuli may be caused by an increase in the production of an enzyme known as ATP, which can be induced by the use of LLLT [12].

On days 1-3 (p=0.000), 5 (p=0.000), and 10 (p=0.000), the size of the ulcer in the test group were significantly reduced. Twenty-eight percent of subjects showed a reduction in ulcer size on day 3, which further increased to 73.19% of subjects by day 5, while on day 10, 100% of subjects were free of ulcers. Aggarwal H et al. [12], Babu et al. [7], and Jijin et al. [20] (810 nm) also found LLLT to be effective in reducing the ulcer size in 83.2% of subjects by the third day which was similar to the present study.

Wounded tissue may be treated with lasers because of their ability to promote wound closure by stimulating the production of basic fibroblast growth factor and fibroblast differentiation into myofibroblasts [21].

Inflammation may be seen as erythema forming around the aphthous ulcer. The shrinkage of the erythema is an indication that the healing process is going as planned. On days 1-3 (p=0.000), 5 (p=0.000), and 10 (p=0.001), there was a substantial decrease in the erythema of ulcers in the test group. Further, all subjects showed lesions surrounded by an erythematous halo on the first visit but after LLLT application there was a significant reduction in erythema in 28.6% of subjects on day 3, in 60.7% of subjects by day 5, and in 92.9% subjects by day 10. Salman H et al. [5] showed similar results as the present study. The presence of erythema around the ulcer decreased from 95.2% in the first visit to 25% after two doses of laser irradiation.

Optically activated suppressor T-lymphocytes were shown to be able to block B-lymphocytes from producing antibodies, which subsequently led to reduced inflammation [18].

Epithelialization is a sign of healing. In the present study, in the test group, there was a significant improvement in the healing of ulcers evaluated from day 3 to day 5 (p=0.000) and day 10 (p=0.000). After LLLT application, ulcer healing was evaluated, and there was a significant improvement in 7.1% of subjects by day 3. Further, by day 5, 42.9% of subjects and 92.9% of subjects by day 10 showed an improvement in the healing of the lesion. Lalabonova H et al., Bladowski et al., and De Souza T et al. showed similar results as compared to the present study. After four to five days of LLLT, 75.6% of patients showed complete epithelialization. Lasers employ precise wavelengths of light, ranging from red to near-infrared, to promote and enhance tissue repair.

An increase in the local tissue blood flow and dilatation of the capillaries are two side effects of LLLT. The photochemical, photophysical, and photobiological impacts of LLLT on tissues are not related to heating; rather, they are the result of the proper dose of photon energy from the LLLT. Mast cells are activated, ATP is produced, and lymphocytes are stimulated. These pro-inflammatory cytokines and leukocytes infiltrate tissues when mast cells become activated. LLLT may play a significant role in promoting wound healing in the oral cavity [7].

TA 0.1% gel

In the present study, the control group (TA 0.1%) showed a gradual reduction in pain score from 35.2% (p=0.000) to 82.3% (p=0.000) within five days, while 98.4% of subjects showed a reduction in pain on day 10 (p=0.007). Unur M et al. [22], Deshmukh and Bagewadi [23], Koray et al. [24], and Miles DA et al. [25] found a similar reduction in pain score within seven days. When TA was used in the first three days of therapy, 20% of the patients reported an improvement in pain [26].

There was a gradual reduction in the size of ulcers in 24.7% of subjects from day 1 to day 3 (p=0.000) and 70% of subjects (p=0.000) by day 5 while complete healing of the ulcer was seen in 100% (p=0.000) by day 10 in the control group. Mehdipour M et al. [27], Deshmukh and Bagewadi [23], and Ofluoglu et al. [28] concluded that the size of ulcer can be successfully managed by using TA, within five days, which is similar to our study.

Edema of ulcers was significantly reduced in the control group between days 1 and 3 (p=0.001), day 5 (p=0.000), and day 10 (p=0.000). All subjects showed lesions surrounded by an erythematous halo on the first visit but after TA gel application there was a significant reduction in erythema in 3.8% of subjects by day 3, in 50% of subjects by day 5, and in 96.2% of subjects by day 10. Salman H et al. [5] suggested TA to be an alternative treatment for RAS. The presence of erythema around the ulcer decreased from 61.5% to 7.7% in the third visit of the subjects.

Vasoconstriction is a major mechanism of action for TA (Kenalog), which may have an immediate or atypical effect by lowering catecholamine, prostaglandin, or receptor levels in specific cell locations. Because of the reduced and increased adherence of white blood cells to the thin endothelium, TA's (Kenalog's) calming effects may also be due to an impedance with the flow of polymorph atomic leukocytes via the fine dividers of the body. Lymphocyte and macrophage activity is also reduced by this medicine, which has soothing effects by reducing the activity of lymphokine molecules. Cell membrane porousness is reduced, toxins or lysosomal proteins arrive more slowly, and other chemical arbiters are hindered or delivered incorrectly throughout the fiery cycle. Each of these arbiters contributes regularly so that vascular porousness and the subsequent alterations such as edema, leukocyte movement, and fibrin statement might increase [29].

After the application of TA gel in the control group, there was a significant improvement in the healing of ulcers evaluated from day 3 to day 5 (p=0.000), and day 10 (p=0.000). On the first visit, 26.9% of subjects in the control group showed a marked improvement by day 3. This increased to 38.5% subjects by day 5 and 96.2% subjects by day 10. The healing of the lesion was seen; however, the findings were insignificant (p=0.096 and p=0.597). Unur M et al. discovered that 64% of individuals exhibited significant improvement by day 7, while ulcer healing was visible by day 5 in the current investigation. M. Unur and colleagues showed that the ulcer healed more quickly following therapy with dexamethasone than with TA. A similar result was found by Gowharyaqub et al. [30] when a comparison was done between topical doxycycline hyclate and a single application of TA.

Glucocorticoids, such as triamcinolone, have anti-inflammatory and anti-proliferative properties when applied to the skin. By binding to certain deoxyribonucleic acid (DNA) response regions in the nucleus and recruiting co-activator proteins, dimers may upregulate gene transcription, but they can also oppose other transcription factor functions [5].

Comparison of control and test groups

In the present study, we compared the test and control groups to evaluate pain, ulcer size, erythema, and healing of ulcers. We found that by day 3, a slightly higher percentage, 42.3%, of subjects showed a reduction in pain in the test group as compared to 35.2% (p=0.798) in the control group, and by day 5 this increased to 85% in the test group and 82.3% in the control group (p=0.853) with complete regression in both groups by day 10 (p=0.940). Thus, LLLT showed a slightly better reduction in pain level by day 3 when compared with topical steroids. de Souza et al. showed similar results as in the present study. Mir M et al. found that laser pen and triamcinolone in orabase ointment have similar results in reducing the pain level in RAS, which was similar to the present study observed on day 10.

In the present study, when the size of the ulcer was evaluated, a slightly higher number, 28%, of subjects showed a reduction by day 3 in the test group as compared to 24.7% of subjects (p=0.651) in the control group. There was a further reduction in the ulcer size on day 5 in 73.1% of the test group and 70% of the control group (p=0.967) subjects, and the ulcer completely healed by day 10. There was a significant change in the size of ulcers in both groups. Thus, the laser shows comparable healing to topical application of TA. Salman H et al. [5] showed similar results as compared to the present study.

We found a statistically significant difference (p=0.13) by day 3, with 28.6% of test subjects free of erythema compared to only 3.8% of control subjects, and by day 5, we found a statistically significant difference (p=0.43) between the two groups, with 60.7% of the test group subjects free of erythema compared to 50% of the control group subjects. Salman H et al. [5] found comparable findings to the current research when erythema was examined, although they found no significant correlation between the test and the control groups. While Lalabonova et al. observed that the erythema was completely handled in individuals treated with LLLT after the first session, it did not alter in any of the patients managed with steroids, which was not comparable to the current investigation.

In the present study, when a comparison was done between the test group and the control group for ulcer healing, we found that a significantly higher percentage of test subjects (p=0.15) by day 3, 7.1%, reported healing while none of the control subjects showed healing of the ulcer. Similarly on day 5, a significantly higher percentage of subjects (p=0.24) in the test group, 42.9%, showed healing of ulcers as compared to 38.5% in the control group. Souza TOFD et al. [18] found similar results as in the present study. In contrast, Salman H et al. [5] found no significant difference in the laser and steroid groups.

The limitations of the study include the use of a limited sample size of subjects, and bias can be developed in the results due to pain analysis on a visual scale where the patient's perception of the pain can be different based on the patient's pain threshold. Long-term follow-up of the patients can be done in the future studies.

## Conclusions

LLLT as a new-age therapy for the treatment of various oral conditions has been utilized in recent years. This therapy is a non-invasive way for clinicians to control many pain- and burning-related lesions in the oral cavity. Thus, to conclude, to achieve a significant reduction in the size of the ulcers, better healing, erythema reduction, and lesser recurrence rates, LLLT can be utilized. The conventional treatments can also be used but being a bit better in pain management the use of lasers can bring a good deal of relief to the subjects and hence can be a good alternative.
